# Erythropoietin Does Not Enhance Skeletal Muscle Protein Synthesis Following Exercise in Young and Older Adults

**DOI:** 10.3389/fphys.2016.00292

**Published:** 2016-07-08

**Authors:** Séverine Lamon, Evelyn Zacharewicz, Emily Arentson-Lantz, Paul A. Della Gatta, Lobna Ghobrial, Frederico Gerlinger-Romero, Andrew Garnham, Douglas Paddon-Jones, Aaron P. Russell

**Affiliations:** ^1^Institute for Physical Activity and Nutrition, School of Exercise and Nutrition Sciences, Deakin UniversityGeelong, VIC, Australia; ^2^Department of Nutrition and Metabolism, University of Texas Medical BranchGalveston, TX, USA

**Keywords:** aging, anabolic signaling, erythropoietin, muscle protein synthesis, resistance exercise

## Abstract

**Purpose:** Erythropoietin (EPO) is a renal cytokine that is primarily involved in hematopoiesis while also playing a role in non-hematopoietic tissues expressing the EPO-receptor (EPOR). The EPOR is present in human skeletal muscle. In mouse skeletal muscle, EPO stimulation can activate the AKT serine/threonine kinase 1 (AKT) signaling pathway, the main positive regulator of muscle protein synthesis. We hypothesized that a single intravenous EPO injection combined with acute resistance exercise would have a synergistic effect on skeletal muscle protein synthesis via activation of the AKT pathway.

**Methods:** Ten young (24.2 ± 0.9 years) and 10 older (66.6 ± 1.1 years) healthy subjects received a primed, constant infusion of [ring-_13_C^6^] L-phenylalanine and a single injection of 10,000 IU epoetin-beta or placebo in a double-blind randomized, cross-over design. 2 h after the injection, the subjects completed an acute bout of leg extension resistance exercise to stimulate skeletal muscle protein synthesis.

**Results:** Significant interaction effects in the phosphorylation levels of the members of the AKT signaling pathway indicated a differential activation of protein synthesis signaling in older subjects when compared to young subjects. However, EPO offered no synergistic effect on vastus lateralis mixed muscle protein synthesis rate in young or older subjects.

**Conclusions:** Despite its ability to activate the AKT pathway in skeletal muscle, an acute EPO injection had no additive or synergistic effect on the exercise-induced activation of muscle protein synthesis or muscle protein synthesis signaling pathways.

## Introduction

Erythropoietin (EPO) is a glycoproteic hormone that is primarily synthetized in the peritubular fibroblast-like cells of the renal cortex (Maxwell et al., 1993, 1997). EPO is produced in response to tissue hypoxia; a process that results in a larger number of circulating erythrocytes and a better oxygenation of the active tissues (Jelkmann, 2004, 2011; Noguchi et al., 2008). Beyond EPO's regulatory function in haematopoiesis and well documented abuse by endurance athletes (Lamon et al., 2010), the EPO-receptor (EPOR) is expressed in several other tissues including endothelial, neural, cardiovascular and muscle tissue (Noguchi et al., 2008). EPO plays various roles in extra-hematopoietic tissues, including protection against oxidative stress (Zaman et al., 1999) and neovascularization (Yasuda et al., 1998). As the EPO molecule binds to its receptor, it induces a conformational change that activates the EPOR (Miura et al., 1991; Dusanter-Fourt et al., 1992) via the phosphorylation of the JAK2 protein (Constantinescu et al., 1999; Remy et al., 1999). EPOR activation in turn triggers STAT5 phosphorylation, which enters the nucleus and activates the transcription of a subset of EPO target genes (Damen et al., 1995; Pallard et al., 1995; Penta and Sawyer, 1995; Klingmuller et al., 1996; Quelle et al., 1996). Alternatively, in interleukin-3-dependent cell lines characterized by high levels of EPOR, EPO-induced JAK2 activation also appears to regulate the phosphatidylinositol 3-kinase (PI3)/AKT (AKT) pathway and the Ras/mitogen-activated kinase (MAPK) signaling pathway (Damen et al., 1993; Miura et al., 1994; Constantinescu et al., 1999; Fisher, 2003). In addition, EPO can activate AKT in extra-hematopoietic tissues including cardiomyocytes (Tramontano et al., 2003), endothelial cells (Elayappan et al., 2009) as well as in the skeletal muscle of mouse overexpressing the EPO protein (Hojman et al., 2009).

The EPOR protein is expressed in human skeletal muscle tissue (Lamon et al., 2014). Skeletal muscle accounts for about 40% of human body mass, houses 50–75% of the body's total protein pool, and is dynamically regulated by the balance between muscle protein synthesis and degradation (Eley and Tisdale, 2007). Maintaining this equilibrium is critical to prevent muscle atrophy and to preserve metabolic health (Rasmussen and Phillips, 2003). However, by the age of 50, age-related muscle wasting can claim as much as 10% of an adult's muscle mass, with a further average reduction of 1% per year thereafter (Dorrens and Rennie, 2003; Phillips, 2012). AKT signaling is an important molecular switch that positively regulates skeletal muscle hypertrophy in myotubes (Rommel et al., 2001) and human muscle tissue (Léger et al., 2006; Sandri et al., 2013; Schiaffino et al., 2013) by enhancing protein synthesis and inhibiting protein degradation pathways. AKT signaling can be activated by various cellular signals including resistance exercise (Léger et al., 2006), a potent anabolic stimulus that promotes muscle protein synthesis and hypertrophy (Marcotte et al., 2015). This anabolic response to resistance exercise is often attenuated in the elderly, who also display reduced phosphorylation of the members of the AKT signaling pathway following exercise (Dennis et al., 2008; Kumar et al., 2009).

Several studies have examined changes in human muscle tissue following acute or chronic systemic injection of recombinant human EPO. A single systemic injection of EPO increased the mRNA levels of the myogenic regulatory factor *MYF6* (formerly MRF-4) 10 h after injection (Lundby et al., 2008b), suggesting a role for EPO and its receptor in muscle development or remodeling. These findings were supported by a recent study showing that 10 weeks of EPO treatment increased PAX7 and MYOD1 content in human satellite cells (Hoedt et al., 2015). An early study on patients with chronic renal failure treated with EPO reported an increase in type I muscle fiber diameter and in muscle glycogen content when compared to baseline levels (Davenport et al., 1993). Finally, muscle mitochondrial oxidative phosphorylation and maximal electron chain transport capacity were enhanced following 8 weeks of EPO treatment in healthy humans (Plenge et al., 2012).

Previous studies have shown that EPO can activate AKT in extra-hematopoietic tissues, including mouse skeletal muscle (Hojman et al., 2009). We hypothesize that a single EPO injection, when combined with acute resistance exercise, will have a synergistic effect on the exercise-induced activation of muscle protein synthesis by activating the AKT pathway in human skeletal muscle. A secondary hypothesis is that if EPO triggers an anabolic response in skeletal muscle, it may be able to rescue a potential reduction in exercise-induced activation of protein synthesis in the older participants.

## Materials and methods

### Subjects

Subject characteristics have been previously described (Stefanetti et al., 2014). Briefly, 10 healthy young (18–30 years old) and 10 healthy older (60–75 years old) males gave their informed consent to participate in the study and agreed to muscle biopsies, physiological testing, and anthropometric measurements. The subjects were physically active but had not participated in a resistance training program or regularly consumed protein supplements for at least 6 months. No subject had a history of anabolic hormone use. The study was approved by the Deakin University Human Research Committee (#2011-043) in accordance to the Declaration of Helsinki (2008) (http://www.wma.net/en/30publications/10policies/b3/).

### Experimental design

**Figure 1:**
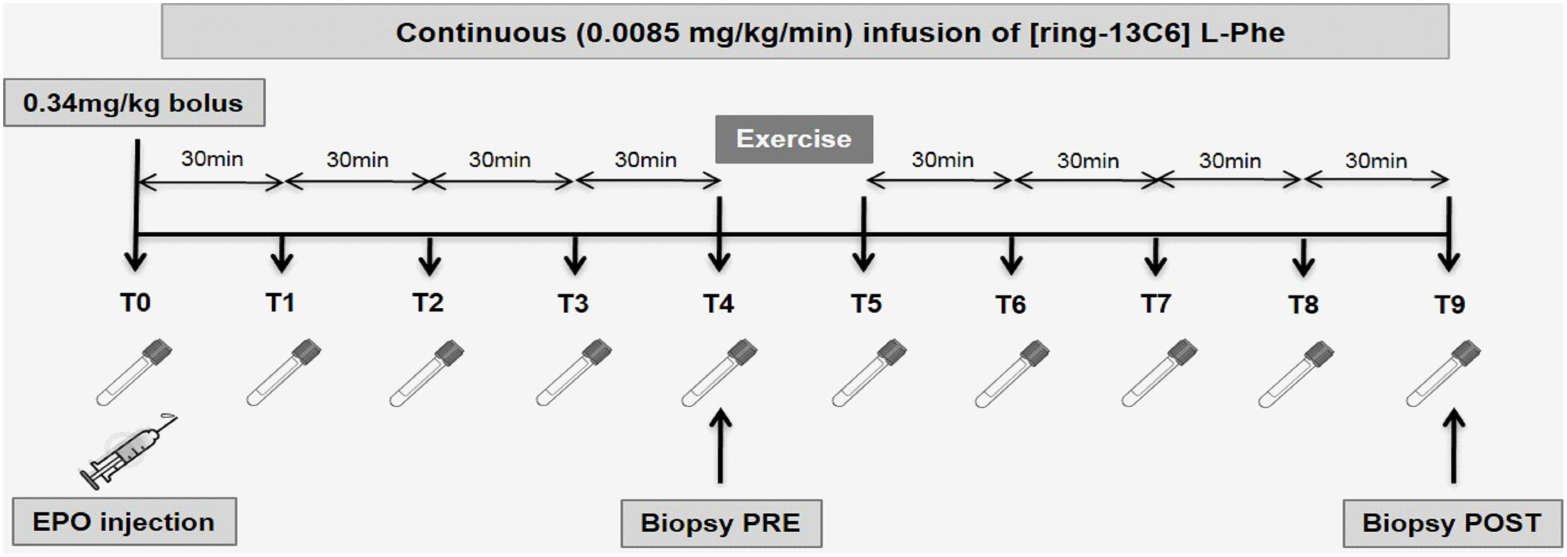
**Experimental timeline of the study**. At time 0, the subjects received a 10,000 IU intra-venous injection of epoetin beta (Recormon®) or a placebo. The exercise bout consisted of 3 sets of 14 repetitions of a leg press exercise at an intensity of 60% of the individual 1 repetition maximum (1RM). Muscle biopsies were collected before and 2 h after exercise. Venous blood samples were collected every 30 min for the whole duration of the study.

Subjects abstained from strenuous exercise, caffeine and alcohol consumption for 24 h prior to the trial. On the night before the trial, the subjects consumed a standardized meal containing 20% fat, 14% protein and 66% carbohydrate. After an overnight fast, the subjects reported to the laboratory at 7 a.m. An 18-gauge cannulae was inserted into the antecubital vein of their left arm for blood sampling and for the injection of 10,000 IU recombinant human EPO (Recormon®, epoetin beta, Roche Pharmaceuticals, Dee Why, NSW) or a saline control (placebo) in a double-blind randomized, cross-over design. This dose represents a compromise between the doses administered to treat patients with renal insufficiencies and the doses reported in doping practices. Another cannulae was inserted in their right arm for a 5 h primed (0.34 mg/kg), continuous infusion of [ring-^13^C_6_] L-phenylalanine (0.0085 mg/kg/min; Cambridge Isotope Laboratories, Tewksbury, MA). The subjects rested in the supine position for 2 h prior to the sampling of the 1st muscle biopsy. Biopsies were obtained from the *vastus lateralis* muscle using 5 mm Bergstrom needle and standard technique, as previously described (Stefanetti et al., 2014). Immediately after the muscle biopsy, subjects completed a 3-min light cycling warm-up followed by an acute bout of 2-leg extension exercise. The exercise protocol consisted of 3 sets of 14 repetitions at 60% of maximal voluntary contraction (60% 1 RM) with 2-min recovery between sets. The subjects then completed a 3-min light cycling cool-down, and remained supine and at rest thereafter. 2 h post-exercise, a second muscle biopsy was taken from the contralateral leg to avoid any local effect of the pre-exercise biopsy. Venous blood samples were collected every 30 min for a 5 h period. Figure 1 depicts the experimental timeline of the study. A minimum of 4 weeks after the trial, the subjects returned to the laboratory and repeated the experiment but this time swapping the treatment for either EPO or the saline control. Dual-Energy X-Ray (DXA) Absorptiometry Scan (Lunar Prodigy, GE Lunar Corp., Madison, WI) was used to assess total body composition, regional (arms and legs) body composition (lean mass, fat mass and percentage body fat), as well as lumbar spine (L1–L4) and proximal femur (femoral neck and total hip) areal bone density. Preliminary testing procedures have been described elsewhere (Stefanetti et al., 2014).

### Protein extraction and western blotting

The protein extraction and western blotting methods have been described elsewhere (Stefanetti et al., 2014) with the following modifications. Equal amount of protein was separated on a 4–12% NuPAGE® Novex Bis-Tris Gel (Life Technologies, Mulgrave, VIC) in NuPAGE® SDS MOPS Running Buffer (Life Technologies) and then transferred onto Immobilon^−^FL PVDF membranes (Millipore, Billerica, MA). For phospho-GSK-3β, equal amount of protein was diluted using a 3X SDS solution separated and run on 18-well 4–12% gradient Criterion SDS-PAGE gels (Bio-Rad Laboratories, Hercules, CA) and then transferred onto Immobilon^−^P PVDF membranes (Millipore). All membranes where then blocked in 5% BSA/PBS. Membranes were incubated at 4⋅C in the following primary antibodies diluted 1:1000 in 5% BSA/PBS: phospho-AKT^*Ser*473^ (CS #9271), phospho-4E-BP1^*Thr*37∕46^ (CS #2855), phospho-p70-S6K^*Thr*389^ (CS #9234), phospho-p44/42MAPK^*Thr*202∕*Tyr*204^ (CS #9101), phospho-GSK-3β^*Ser*9^ (CS #9336; Cell Signaling Technologies, Arundel, QLD). Following overnight incubation, the membranes were washed and incubated for 1 h with a goat anti-rabbit IgG antibody labeled with an infrared-fluorescent 800 nm dye (Alexa Fluor® 800, Life Technologies) diluted 1:10,000 in a buffer containing PBS and Odyssey® blocking buffer (LI-COR Biosciences, Lincoln, NE) at a 1:1 ratio and 0.01% SDS or an anti-rabbit IgG (H+L) DyLight 800 Conjugate (Genesearch diluted 1:10,000 in a buffer containing 5% BSA. After washing, the proteins were exposed on an Odyssey® Infrared Imaging System (LI-COR Biosciences) and individual protein band optical densities were determined using the Odyssey® Infrared Imaging System software. All blots were normalized to the GAPDH protein (G8795; Sigma-Aldrich, Sydney, NSW). For phospho-GSK-3β, the proteins were exposed on a Gel Doc imaging system (Bio-Rad Laboratories). Individual protein band optical densities were determined using the Image Lab software (Bio-Rad Laboratories) and all blots were normalized to the total protein load.

### Isotopic enrichment in plasma

Venous blood samples were collected in heparin-tubes, manually inverted and immediately centrifuged for 15 min at 13,000 RPM at 4⋅C. The supernatant (plasma) was then isolated and frozen at −80⋅C for further analysis. After thawing, plasma was precipitated using an equal volume of 15% sulfosalicylic acid (SSA) solution and centrifuged for 20 min at 13,000 RPM at 4⋅C. Blood amino acids were extracted from 500 mL of supernatant by cation exchange chromatography (Dowex AG 50W–8X, 100–200 mesh H+ form; Bio-Rad Laboratories). Phenylalanine enrichments were determined by gas chromatography–mass spectrometry (GC–MS) using the tertbutyldimethylsilyl derivative with electron impact ionization as described previously (Freischmidt et al., 2015). Ions 336 and 342 were monitored.

### Isotopic enrichment in muscle proteins

A 30 mg piece of muscle biopsy was used for isolation of mixed muscle bound (myofibrillar) and intracellular protein fractions. Briefly, bound muscle proteins were extracted in perchloric acid and hydrolysed using 6N hydrochloric acid (110⋅C for 24 h). Isotopic enrichments of [ring-_13_C^6^]-L-phenylalanine in tissue fluid (intracellular fraction) were used as a precursor pool for the calculation of the fractional synthesis rate (FSR). Total muscle phenylalanine was isolated using cation exchange chromatography (50W-8X, 200–400 mesh H+ form; Bio-Rad Laboratories). Amino acids were eluted in 8 mL of 2N ammonium hydroxide and dried under vacuum. Muscle intracellular and bound protein [ring-_13_C^6^]-L-phenylalanine enrichments were determined by GC-MS with electron impact ionization using the tert-butyldimethylsilyl derivative. Ions 238 and 240 were monitored for bound protein enrichments; ions 336 and 342 were monitored for intracellular enrichments as described previously (Freischmidt et al., 2015). Mixed muscle protein FSR (%/h) was calculated by measuring the direct incorporation of [ring-_13_C^6^]-L-phenylalanine by using the precursor-product model (Chang et al., 2010; Mei et al., 2012; Mestdagh et al., 2014):

$$\begin{array}{l} FSR=\frac{EP2-EP1}{\left(Emxt\right)}x60x100 \end{array}$$

where *EP1* and *EP2* are the bound enrichments of [ring-_13_C^6^]-L-phenylalanine for the two muscle biopsies, *Em* is the mean enrichment of [ring-_13_C^6^]-L-phenylalanine in the muscle intracellular pool, and *t* is the time interval (min) between the two biopsies.

### Statistical methods

All data are reported as mean ± SEM. Paired *t*-tests or the mixed-model 2-way analysis of variance (ANOVA) were used to compare group means. Diagnostic plots of residuals and fitted values were checked to ensure homogeneity of variance (a key assumption for ANOVA). Consequently, all protein expression data was log10-transformed and analyses were conducted on these transformed scales. The least significant difference (LSD) test was used to compare pairs of means. The significance levels for the *F*-tests in the *t*-tests and ANOVA and the LSD tests were set at *p* < 0.05.

## Results

### Subjects' demographics

Subjects' demographics has been reported in a previously published manuscript (Stefanetti et al., 2014). Briefly, the two subject groups were respectively aged 24.2 ± 0.9 y.o. and 66.6 ± 1.1 y.o. and presented no significant difference in body mass (young: 73.8 ± 3.6 kg, older: 83.4 ± 7.1 kg), fat mass (young: 13.6 ± 2.7 kg, older: 19.5 ± 3.9 kg), lean mass (young: 57.9 ± 1.9 kg, older: 60.2 ± 3.3 kg), lean/total body mass ratio (young: 0.8 ± 0.02, older: 0.7 ± 0.02) and maximal voluntary contraction (1RM; young: 98.2 ± 7.0 kg, older: 80.9 ± 7.2 kg).

### Muscle protein synthesis

**Figure 2:**
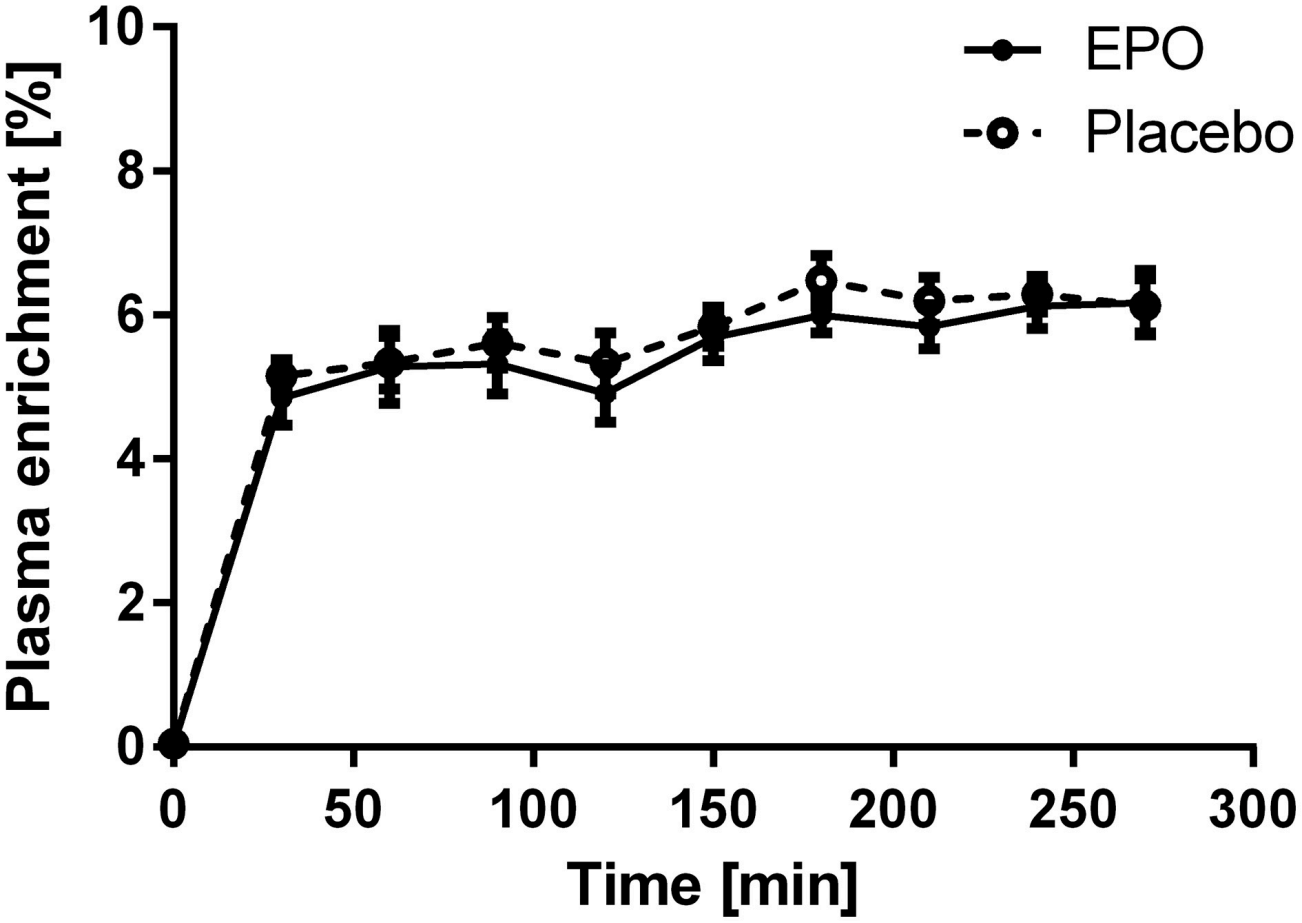
**Plasma enrichment of [ring-^**13**^C_**6**_] L-phenylalanine during the study; enrichments were averaged within age groups. EPO, ***n*** = 6; Placebo, ***n*** = 6**.

**Figure 3:**
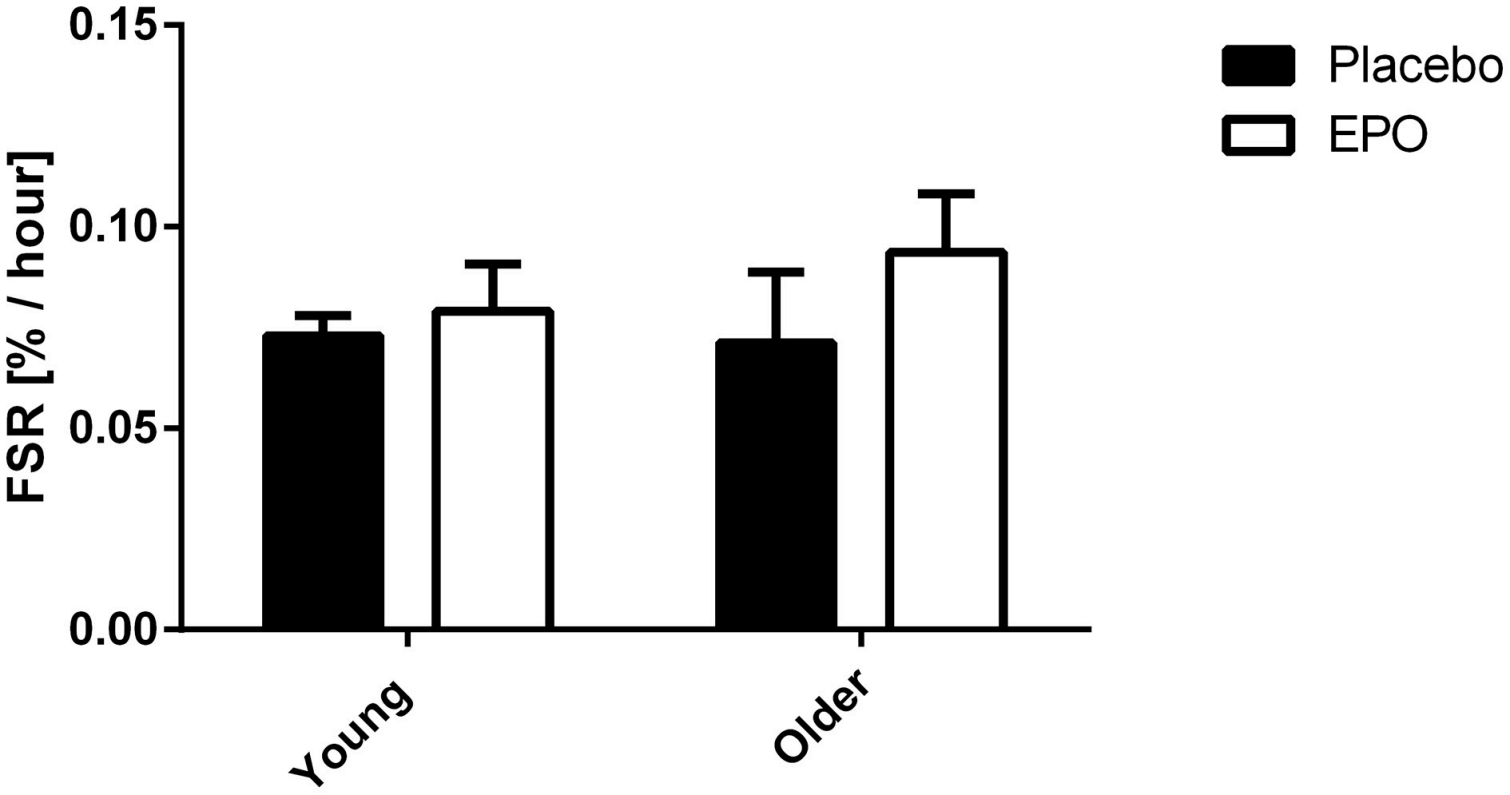
**Mixed muscle fractional synthesis rate measured in young (24.2 ± 0.9 y.o.) and older (66.6 ± 1.1 y.o.) subjects between 0 and 2 h post exercise**. There was no significant difference due to treatment, age or a combination of both factors.

Subjects remained in isotopic steady-state throughout the infusion protocol, with no difference due to EPO treatment (Figure 2). EPO treatment did not influence FSR in young or older subjects. The average FSR for all subjects was 0.08% per hour and no difference existed between young and older subjects (Figure 3).

### Protein expression of the members of the protein synthesis pathways

**Figure 4:**
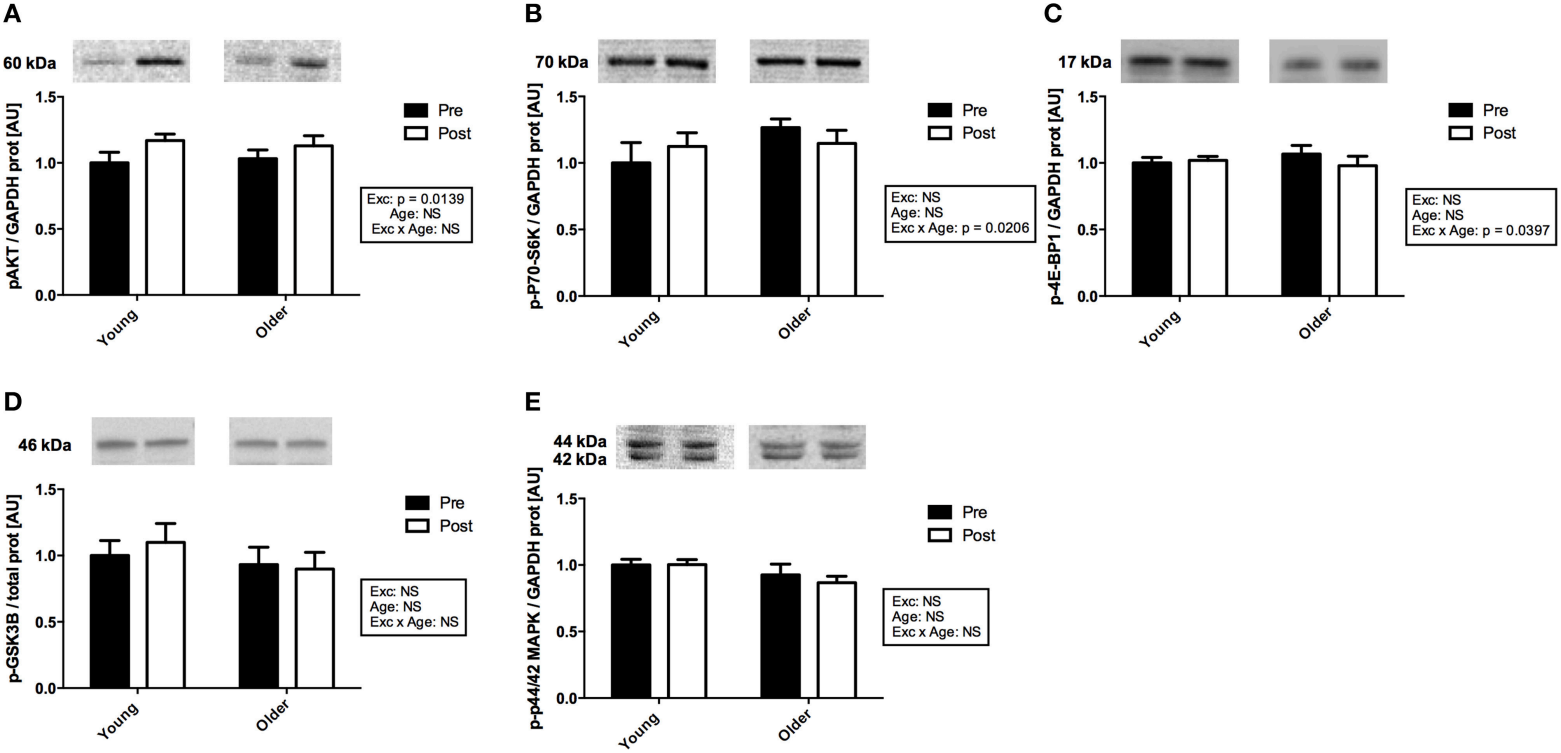
**Activation of the members of the protein synthesis signaling pathways with exercise in muscle from young and older subjects**. **(A)**. Representative immunoblots of phospho-AKT^Ser473^ and corresponding quantification. There was a significant main effect of exercise (*p* = 0.0139). **(B)**. Representative immunoblots of phospho-4E-BP1^Thr37∕46^ and corresponding quantification. There was a significant age × exercise interaction (*p* = 0.0206). **(C)**. Representative immunoblots of phospho-p70-S6K^Thr389^ and corresponding quantification. There was a significant age x exercise interaction (*p* < 0.0397). **(D)**. Representative immunoblots of phospho-GSK3B^Ser9^ and corresponding quantification. **(E)**. Representative immunoblots of phospho-p44/42 MAPK ^Thr202∕Tyr204^ and corresponding quantification. Note that non-contiguous gel lanes are demarcated by white spaces; no adjustment to digital images do alter the information contained therein.

The phosphorylation levels of the members of the AKT and the MAPK signaling pathways were measured in muscle tissue obtained pre- and post-exercise. As no difference was detected between the two treatment groups (EPO vs. placebo) for any of the measured targets, the protein data were pooled for further analysis. Two hour following exercise, there was a main effect of exercise on AKT phosphorylation levels (*p* < 0.05; Figure 4A). It should however be noticed that, when individual *t*-tests were run, this difference appeared significant in the young cohort only (Zacharewicz et al., 2014). No main effect of exercise was detected in the protein levels of phospho-p70-S6K and phospho-4E-BP1, however an age x exercise interaction was apparent for both (*p* < 0.05; Figures 4B,C). Associated *post-hoc* tests were not significant. Finally, no change due to age or exercise was observed on phospho-GSK3B or phospho-p44/42MAPK protein levels (Figures 4D,E).

## Discussion

Resistance exercise is a potent activator of anabolic pathways in skeletal muscle (Marcotte et al., 2015). Here we investigated for the first time the effect of an acute systemic EPO injection on muscle protein synthesis following a single protein synthesis stimulating exercise bout. We hypothesized that EPO may require a concurrent anabolic stimulus to exert its effects in muscle and as a result, that exercise and EPO may have a synergistic effect on muscle protein synthesis. However, we report that a single EPO injection does not alter muscle protein synthesis rates or signaling following an acute resistance exercise bout in young or older subjects.

Our study is the first to evaluate the potential synergistic effect of acute exercise and EPO on human skeletal muscle FSR. Christensen et al. (2012b) have previously evaluated the effects of a single systemic injection of 400 IU/kg EPO on whole body and forearm protein metabolism, measured as carbamide, phenylalanine, and tyrosine fluxes, in young males in a resting situation only. These authors reported no effect of EPO injection on whole body and forearm protein turnover at rest. Another study showed that EPO treatment increased type I muscle fiber diameter in patients with chronic renal failure when compared to pre-treatment levels (Davenport et al., 1993), although 14 weeks of EPO treatment had no effect on muscle fiber hypertrophy in young males (Lundby et al., 2008a). A positive effect of EPO on muscle hypertrophy was however supported by rodent data showing that, in a mouse model where the *Epo* gene was electro transferred, resulting in supra-physiological EPO levels, mice displayed significant leg muscle hypertrophy when compared to their contralateral non-transfected leg and to control mice (Hojman et al., 2009). Taken together, these results suggest that extra-physiological levels of EPO, achieved by long-term high-dose treatment or gene modification, may be necessary to observe an effect of EPO on skeletal muscle protein accretion and hypertrophy.

The mixed muscle FSR we observed 2 h following a bout of resistance exercise were consistent with the values obtained 1-and 2-h following a similar exercise regime (Kumar et al., 2009). No effect of age was observed. An average lower protein synthesis response in elderly subjects when compared to younger subjects has only been observed when measured 1–2 h post-exercise (average response to 60–90% 1RM intensities), but no difference was reported when measured 0–1 h post-exercise. In addition, no breakdown of the response induced by the different exercise intensities was provided, suggesting that an age effect may only occur at higher exercise intensities (Kumar et al., 2009). In addition, Fry et al. did not report any effect of age when FSR was measured 0–3 h post a resistance exercise bout consisting of eight sets of 10 repetitions at 70% 1RM, leading to the hypothesis that a decrease in protein synthesis activation in older subjects may only be observed after a certain time delay (Fry et al., 2011).

Upstream from the protein synthesis process itself, the phosphorylation levels of the AKT protein significantly increased 2 h post-exercise when compared to baseline. In addition, there was a combined effect of age and exercise on the phosphorylation levels of the p70-S6K and 4E-BP1 proteins suggesting that muscle protein synthesis signaling is impaired in older when compared to young subjects (Dennis et al., 2008; Kumar et al., 2009), although this may only translate to the FSR at a later time point. While *post-hoc* tests did not reveal significant differences within age group means for phospho-p70-S6K and phospho-4E-BP1, this might be indicative of these proteins returning to their baseline levels 2 h post-exercise. Failure to observe phosphorylation changes in p70-S6K and 4E-BP1 2 h following resistance exercise has been reported before, independent of protein synthesis activation (Kumar et al., 2009). Of interest, in a previous publication we reported no age-related difference in the regulation of the members of the ubiquitin proteasome pathway following exercise in the same cohort (Stefanetti et al., 2014), suggesting that in contrast to protein synthesis, protein degradation signaling is not affected by age.

Consistent with the lack of effect on muscle protein synthesis, EPO did not further activate AKT signaling or MAPK signaling following exercise. In human skeletal muscle at rest, an acute systemic injection of 400 IU/kg EPO did not alter the baseline phosphorylation levels of AKT, MAPK, STAT5, and NFKB when measured between 1 and 6 h following treatment (Christensen et al., 2012a). We recently reported similar observations in human primary muscle cells treated with EPO and proposed that the EPOR is present, but may not be functional in human muscle cells (Lamon et al., 2014). Still, EPO has shown effects on skeletal muscle tissue, which may therefore not be via the EPOR but via a surrogate muscle specific or non-specific pathway. In healthy humans, 8 weeks of EPO treatment enhanced muscle mitochondrial oxidative phosphorylation and maximal electron transport capacity (Plenge et al., 2012). This outcome is in line with observations made in earlier rodent studies (Cayla et al., 2008; Hojman et al., 2009) but may result from hyperoxia augmenting muscle oxidative capacity (Ploutz-Snyder et al., 1996), rather than from a direct effect of EPO in muscle. EPO administration increased whole-body energy expenditure (Christensen et al., 2012b) and it was recently suggested that the mechanism may be via an increase in *UCP2* mRNA levels in muscle (Christensen et al., 2013). Finally, several studies suggest that EPO plays a role in skeletal muscle regeneration in humans. A recent study reported that in human muscle tissue, EPO may assist in stimulating the myogenic fate in satellite cells (Hoedt et al., 2015). In addition, patients suffering Friedrich ataxia displayed improved motor function when treated with EPO (Nachbauer et al., 2012). A role for EPO in improving muscle regeneration and strength following different types of injuries has also been reported in different rodent studies (Kao et al., 2007; Rotter et al., 2008, 2012; Jia et al., 2012). Altogether, these results suggest that the effects of acute or chronic EPO injection on human skeletal muscle may not be exerted by a direct activation of the EPOR, but rather be the consequence of an enhanced oxygen carrying capacity of the blood. Alternatively, there might be a need to investigate models displaying supra-physiological EPO concentrations (Hojman et al., 2009) or extreme metabolic challenges (Nachbauer et al., 2012) to observe an activation of the EPO-R in skeletal muscle.

Our study is the first one to examine the combined effects of EPO and exercise on muscle protein synthesis and to assess these effects in young and older subjects. State-of-the art radiolabelled tracer technology was used to investigate protein synthesis *in vivo* and further confirmed time discrepancies between the activation of the markers of protein synthesis and the actual functional outcome. While both parameters are contingent, caution should be taken when only reporting changes in marker expression. As it is often the case in human exercise trials, one important limitation of our study is the difference existing between the studied population cohort and the age-matched average population cohort in terms of physiological characteristics and level of fitness. It is commonly acknowledged that the older subjects recruited on a voluntary basis for an exercise trial protocol are not representative of the average elderly population and that their average level of fitness is expected to be higher. This parameter therefore needs to be considered when comparing our results to other studies.

In conclusion, a single bout of resistance exercise activated muscle protein synthesis signaling in young and old skeletal muscle but an acute EPO injection had no synergistic effect on muscle protein synthesis rates or on the activation of the muscle protein synthesis signaling pathways. This suggests that EPO is not able to activate AKT and MAPK signaling in young or older human skeletal muscle. These results refute a potential anabolic role of EPO in human skeletal muscle and contribute to calls to limit its misuse as a doping agent.

## Author contributions

SL and AR designed and funded the study. SL, EZ, EA, PD, LG, and FG collected the data. SL, EZ, and EA analyzed the data. AG collected the human muscle biopsies. DP provided guidance with study design and data analysis. All authors contributed to manuscript writing and edition.

### Conflict of interest statement

The authors declare that the research was conducted in the absence of any commercial or financial relationships that could be construed as a potential conflict of interest.

## References

[B1] Cayla J. L. Maire P. Duvallet A. Wahrmann J. P. (2008). Erythropoietin induces a shift of muscle phenotype from fast glycolytic to slow oxidative. Int. J. Sports Med. 29, 460–465. 10.1055/s-2007-965359 18080952

[B2] Chang K. H. Mestdagh P. Vandesompele J. Kerin M. J. Miller N. (2010). MicroRNA expression profiling to identify and validate reference genes for relative quantification in colorectal cancer. BMC Cancer 10:173. 10.1186/1471-2407-10-173 20429937 PMC2873395

[B3] Christensen B. Lundby C. Jessen N. Nielsen T. S. Vestergaard P. F. Moller N.. (2012a). Evaluation of functional erythropoietin receptor status in skeletal muscle *in vivo*: acute and prolonged studies in healthy human subjects. PLoS ONE 7:e31857. 10.1371/journal.pone.0031857 22384088 PMC3285196

[B4] Christensen B. Nellemann B. Larsen M. S. Thams L. Sieljacks P. Vestergaard P. F.. (2013). Whole body metabolic effects of prolonged endurance training in combination with erythropoietin treatment in humans: a randomized placebo controlled trial. Am. J. Physiol. Endocrinol. Metab. 305, E879–E889. 10.1152/ajpendo.00269.2013 23921143

[B5] Christensen B. Vendelbo M. H. Krusenstjerna-Hafstrom T. Madsen M. Pedersen S. B. Jessen N.. (2012b). Erythropoietin administration acutely stimulates resting energy expenditure in healthy young men. J. Appl. Physiol. 112, 1114–1121. 10.1152/japplphysiol.01391.2011 22241056

[B6] Constantinescu S. N. Ghaffari S. Lodish H. F. (1999). The erythropoietin receptor: structure, activation and intracellular signal transduction. Trends Endocrinol. Metab. 10, 18–23. 10.1016/S1043-2760(98)00101-5 10322390

[B7] Damen J. E. Mui A. L. Puil L. Pawson T. Krystal G. (1993). Phosphatidylinositol 3-kinase associates, via its Src homology 2 domains, with the activated erythropoietin receptor. Blood 81, 3204–3210. 7685197

[B8] Damen J. E. Wakao H. Miyajima A. Krosl J. Humphries R. K. Cutler R. L.. (1995). Tyrosine 343 in the erythropoietin receptor positively regulates erythropoietin-induced cell proliferation and Stat5 activation. EMBO J. 14, 5557–5568. 8521813 10.1002/j.1460-2075.1995.tb00243.x PMC394670

[B9] Davenport A. King R. F. Ironside J. W. Will E. J. Davison A. M. (1993). The effect of treatment with recombinant human erythropoietin on the histological appearance and glycogen content of skeletal muscle in patients with chronic renal failure treated by regular hospital haemodialysis. Nephron 64, 89–94. 10.1159/000187284 8502342

[B10] Dennis R. A. Przybyla B. Gurley C. Kortebein P. M. Simpson P. Sullivan D. H.. (2008). Aging alters gene expression of growth and remodeling factors in human skeletal muscle both at rest and in response to acute resistance exercise. Physiol. Genomics 32, 393–400. 10.1152/physiolgenomics.00191.2007 18073271 PMC6581202

[B11] Dorrens J. Rennie M. J. (2003). Effects of ageing and human whole body and muscle protein turnover. Scand. J. Med. Sci. Sports 13, 26–33. 10.1034/j.1600-0838.2003.00306.x 12535314

[B12] Dusanter-Fourt I. Casadevall N. Lacombe C. Muller O. Billat C. Fischer S.. (1992). Erythropoietin induces the tyrosine phosphorylation of its own receptor in human erythropoietin-responsive cells. J. Biol. Chem. 267, 10670–10675. 1375226

[B13] Elayappan B. Ravinarayannan H. Pasha S. P. Lee K. J. Gurunathan S. (2009). PEDF inhibits VEGF- and EPO- induced angiogenesis in retinal endothelial cells through interruption of PI3K/Akt phosphorylation. Angiogenesis 12, 313–324. 10.1007/s10456-009-9153-5 19657716

[B14] Eley H. L. Tisdale M. J. (2007). Skeletal muscle atrophy, a link between depression of protein synthesis and increase in degradation. J. Biol. Chem. 282, 7087–7097. 10.1074/jbc.M610378200 17213191

[B15] Fisher J. W. (2003). Erythropoietin: physiology and pharmacology update. Exp. Biol. Med. (Maywood). 228, 1–14. 12524467 10.1177/153537020322800101

[B16] Freischmidt A. Muller K. Zondler L. Weydt P. Mayer B. Von Arnim C. A.. (2015). Serum microRNAs in sporadic amyotrophic lateral sclerosis. Neurobiol. Aging 36, 2660.e2615–e2620. 10.1016/j.neurobiolaging.2015.06.003 26142125

[B17] Fry C. S. Drummond M. J. Glynn E. L. Dickinson J. M. Gundermann D. M. Timmerman K. L.. (2011). Aging impairs contraction-induced human skeletal muscle mTORC1 signaling and protein synthesis. Skelet. Muscle 1:11. 10.1186/2044-5040-1-11 21798089 PMC3156634

[B18] Hoedt A. Christensen B. Nellemann B. Mikkelsen U. R. Hansen M. Schjerling P.. (2015). Satellite cell response to erythropoietin treatment and endurance training in healthy young men. J. Physiol. 594, 727–743. 10.1113/JP271333 26607845 PMC5341704

[B19] Hojman P. Brolin C. Gissel H. Brandt C. Zerahn B. Pedersen B. K.. (2009). Erythropoietin over-expression protects against diet-induced obesity in mice through increased fat oxidation in muscles. PLoS ONE 4:e5894. 10.1371/journal.pone.0005894 19521513 PMC2690401

[B20] Jelkmann W. (2004). Molecular biology of erythropoietin. Intern. Med. 43, 649–659. 10.2169/internalmedicine.43.649 15468961

[B21] Jelkmann W. (2011). Regulation of erythropoietin production. J. Physiol. 589, 1251–1258. 10.1113/jphysiol.2010.195057 21078592 PMC3082088

[B22] Jia Y. Suzuki N. Yamamoto M. Gassmann M. Noguchi C. T. (2012). Endogenous erythropoietin signaling facilitates skeletal muscle repair and recovery following pharmacologically induced damage. FASEB J. 26, 2847–2858. 10.1096/fj.11-196618 22490927 PMC3382092

[B23] Kao R. Xenocostas A. Rui T. Yu P. Huang W. Rose J.. (2007). Erythropoietin improves skeletal muscle microcirculation and tissue bioenergetics in a mouse sepsis model. Crit. Care 11:R58. 10.1186/cc5920 17509156 PMC2206412

[B24] Klingmuller U. Bergelson S. Hsiao J. G. Lodish H. F. (1996). Multiple tyrosine residues in the cytosolic domain of the erythropoietin receptor promote activation of STAT5. Proc. Natl. Acad. Sci. U.S.A. 93, 8324–8328. 10.1073/pnas.93.16.8324 8710869 PMC38669

[B25] Kumar V. Selby A. Rankin D. Patel R. Atherton P. Hildebrandt W.. (2009). Age-related differences in the dose-response relationship of muscle protein synthesis to resistance exercise in young and old men. J. Physiol. 587, 211–217. 10.1113/jphysiol.2008.164483 19001042 PMC2670034

[B26] Lamon S. Robinson N. Saugy M. (2010). Procedures for monitoring recombinant erythropoietin and analogs in doping. Endocrinol. Metab. Clin. North Am. 39, 141–154, x. 10.1016/j.ecl.2009.10.004 20122455

[B27] Lamon S. Zacharewicz E. Stephens A. N. Russell A. P. (2014). EPO-receptor is present in mouse C2C12 and human primary skeletal muscle cells but EPO does not influence myogenesis. Physiol. Rep. 2:e00256. 10.1002/phy2.256 24760510 PMC4002236

[B28] Léger B. Cartoni R. Praz M. Lamon S. Dériaz O. Crettenand A.. (2006). Akt signalling through GSK-3beta, mTOR and Foxo1 is involved in human skeletal muscle hypertrophy and atrophy. J. Physiol. 576, 923–933. 10.1113/jphysiol.2006.116715 16916907 PMC1890416

[B29] Lundby C. Hellsten Y. Jensen M. B. Munch A. S. Pilegaard H. (2008a). Erythropoietin receptor in human skeletal muscle and the effects of acute and long-term injections with recombinant human erythropoietin on the skeletal muscle. J. Appl. Physiol. (1985) 104, 1154–1160. 10.1152/japplphysiol.01211.2007 18218911

[B30] Lundby C. Robach P. Boushel R. Thomsen J. J. Rasmussen P. Koskolou M.. (2008b). Does recombinant human Epo increase exercise capacity by means other than augmenting oxygen transport? J. Appl. Physiol. 105, 581–587. 10.1152/japplphysiol.90484.2008 18535134

[B31] Marcotte G. R. West D. W. Baar K. (2015). The molecular basis for load-induced skeletal muscle hypertrophy. Calcif. Tissue Int. 96, 196–210. 10.1007/s00223-014-9925-9 25359125 PMC4809742

[B32] Maxwell P. H. Ferguson D. J. Nicholls L. G. Iredale J. P. Pugh C. W. Johnson M. H.. (1997). Sites of erythropoietin production. Kidney Int. 51, 393–401. 10.1038/ki.1997.52 9027712

[B33] Maxwell P. H. Osmond M. K. Pugh C. W. Heryet A. Nicholls L. G. Tan C. C.. (1993). Identification of the renal erythropoietin-producing cells using transgenic mice. Kidney Int. 44, 1149–1162. 10.1038/ki.1993.362 8264149

[B34] Mei Q. Li X. Meng Y. Wu Z. Guo M. Zhao Y.. (2012). A facile and specific assay for quantifying microRNA by an optimized RT-qPCR approach. PLoS ONE 7:e46890. 10.1371/journal.pone.0046890 23071657 PMC3465266

[B35] Mestdagh P. Hartmann N. Baeriswyl L. Andreasen D. Bernard N. Chen C.. (2014). Evaluation of quantitative miRNA expression platforms in the microRNA quality control (miRQC) study. Nat. Methods 11, 809–815. 10.1038/nmeth.3014 24973947

[B36] Miura O. D'andrea A. Kabat D. Ihle J. N. (1991). Induction of tyrosine phosphorylation by the erythropoietin receptor correlates with mitogenesis. Mol. Cell. Biol. 11, 4895–4902. 10.1128/MCB.11.10.4895 1656216 PMC361458

[B37] Miura Y. Miura O. Ihle J. N. Aoki N. (1994). Activation of the mitogen-activated protein kinase pathway by the erythropoietin receptor. J. Biol. Chem. 269, 29962–29969. 7961995

[B38] Nachbauer W. Boesch S. Reindl M. Eigentler A. Hufler K. Poewe W.. (2012). Skeletal muscle involvement in friedreich ataxia and potential effects of recombinant human erythropoietin administration on muscle regeneration and neovascularization. J. Neuropathol. Exp. Neurol. 71, 708–715. 10.1097/NEN.0b013e31825fed76 22805773

[B39] Noguchi C. T. Wang L. Rogers H. M. Teng R. Jia Y. (2008). Survival and proliferative roles of erythropoietin beyond the erythroid lineage. Expert Rev. Mol. Med. 10:e36. 10.1017/S1462399408000860 19040789 PMC3065109

[B40] Pallard C. Gouilleux F. Charon M. Groner B. Gisselbrecht S. Dusanter-Fourt I. (1995). Interleukin-3, erythropoietin, and prolactin activate a STAT5-like factor in lymphoid cells. J. Biol. Chem. 270, 15942–15945. 10.1074/jbc.270.27.15942 7608147

[B41] Penta K. Sawyer S. T. (1995). Erythropoietin induces the tyrosine phosphorylation, nuclear translocation, and DNA binding of STAT1 and STAT5 in erythroid cells. J. Biol. Chem. 270, 31282–31287. 10.1074/jbc.270.52.31282 8537396

[B42] Phillips S. M. (2012). Nutrient-rich meat proteins in offsetting age-related muscle loss. Meat Sci. 92, 174–178. 10.1016/j.meatsci.2012.04.027 22632883

[B43] Plenge U. Belhage B. Guadalupe-Grau A. Andersen P. R. Lundby C. Dela F.. (2012). Erythropoietin treatment enhances muscle mitochondrial capacity in humans. Front. Physiol. 3:50. 10.3389/fphys.2012.00050 22419911 PMC3299978

[B44] Ploutz-Snyder L. L. Simoneau J. A. Gilders R. M. Staron R. S. Hagerman F. C. (1996). Cardiorespiratory and metabolic adaptations to hyperoxic training. Eur. J. Appl. Physiol. Occup. Physiol. 73, 38–48. 10.1007/BF00262807 8861667

[B45] Quelle F. W. Wang D. Nosaka T. Thierfelder W. E. Stravopodis D. Weinstein Y.. (1996). Erythropoietin induces activation of Stat5 through association with specific tyrosines on the receptor that are not required for a mitogenic response. Mol. Cell. Biol. 16, 1622–1631. 10.1128/MCB.16.4.1622 8657137 PMC231148

[B46] Rasmussen B. B. Phillips S. M. (2003). Contractile and nutritional regulation of human muscle growth. Exerc. Sport Sci. Rev. 31, 127–131. 10.1097/00003677-200307000-00005 12882478

[B47] Remy I. Wilson I. A. Michnick S. W. (1999). Erythropoietin receptor activation by a ligand-induced conformation change. Science 283, 990–993. 10.1126/science.283.5404.990 9974393

[B48] Rommel C. Bodine S. C. Clarke B. A. Rossman R. Nunez L. Stitt T. N.. (2001). Mediation of IGF-1-induced skeletal myotube hypertrophy by PI(3)K/Akt/mTOR and PI(3)K/Akt/GSK3 pathways. Nat. Cell Biol. 3, 1009–1013. 10.1038/ncb1101-1009 11715022

[B49] Rotter R. Kuhn C. Stratos I. Beck M. Mittlmeier T. Vollmar B. (2012). Erythropoietin enhances the regeneration of traumatized tissue after combined muscle-nerve injury. J. Trauma Acute Care Surg. 72, 1567–1575. 10.1097/TA.0b013e318246498f 22695424

[B50] Rotter R. Menshykova M. Winkler T. Matziolis G. Stratos I. Schoen M.. (2008). Erythropoietin improves functional and histological recovery of traumatized skeletal muscle tissue. J. Orthop. Res. 26, 1618–1626. 10.1002/jor.20692 18634017

[B51] Sandri M. Barberi L. Bijlsma A. Y. Blaauw B. Dyar K. A. Milan G.. (2013). Signalling pathways regulating muscle mass in ageing skeletal muscle: the role of the IGF1-Akt-mTOR-FoxO pathway. Biogerontology 14, 303–323. 10.1007/s10522-013-9432-9 23686362

[B52] Schiaffino S. Dyar K. A. Ciciliot S. Blaauw B. Sandri M. (2013). Mechanisms regulating skeletal muscle growth and atrophy. FEBS J. 280, 4294–4314. 10.1111/febs.12253 23517348

[B53] Stefanetti R. J. Zacharewicz E. Della Gatta P. Garnham A. Russell A. P. Lamon S. (2014). Ageing has no effect on the regulation of the ubiquitin proteasome-related genes and proteins following resistance exercise. Front. Physiol. 5:30. 10.3389/fphys.2014.00030 24550841 PMC3907707

[B54] Tramontano A. F. Muniyappa R. Black A. D. Blendea M. C. Cohen I. Deng L.. (2003). Erythropoietin protects cardiac myocytes from hypoxia-induced apoptosis through an Akt-dependent pathway. Biochem. Biophys. Res. Commun. 308, 990–994. 10.1016/S0006-291X(03)01503-1 12927817

[B55] Yasuda Y. Masuda S. Chikuma M. Inoue K. Nagao M. Sasaki R. (1998). Estrogen-dependent production of erythropoietin in uterus and its implication in uterine angiogenesis. J. Biol. Chem. 273, 25381–25387. 10.1074/jbc.273.39.25381 9738005

[B56] Zacharewicz E. Della Gatta P. Reynolds J. Garnham A. Crowley T. Russell A. P.. (2014). Identification of microRNAs linked to regulators of muscle protein synthesis and regeneration in young and old skeletal muscle. PLoS ONE 9:e114009. 10.1371/journal.pone.0114009 25460913 PMC4252069

[B57] Zaman K. Ryu H. Hall D. O'donovan K. Lin K. I. Miller M. P.. (1999). Protection from oxidative stress-induced apoptosis in cortical neuronal cultures by iron chelators is associated with enhanced DNA binding of hypoxia-inducible factor-1 and ATF-1/CREB and increased expression of glycolytic enzymes, p21(waf1/cip1), and erythropoietin. J. Neurosci. 19, 9821–9830. 10559391 10.1523/JNEUROSCI.19-22-09821.1999 PMC6782985

